# The Impact of Glutamatergic Synapse Dysfunction in the Corticothalamocortical Network on Absence Seizure Generation

**DOI:** 10.3389/fnmol.2022.836255

**Published:** 2022-02-14

**Authors:** Beulah Leitch

**Affiliations:** Department of Anatomy, School of Biomedical Sciences, Brain Health Research Centre, University of Otago, Dunedin, New Zealand

**Keywords:** AMPA receptors, excitatory synapses, feed-forward inhibition, absence epilepsy, corticothalamocortical network, DREADD technology

## Abstract

Childhood absence epilepsy (CAE) is the most common pediatric epilepsy affecting 10–18% of all children with epilepsy. It is genetic in origin and the result of dysfunction within the corticothalamocortical (CTC) circuitry. Network dysfunction may arise from multifactorial mechanisms in patients from different genetic backgrounds and thus account for the variability in patient response to currently available anti-epileptic drugs; 30% of children with absence seizures are pharmaco-resistant. This review considers the impact of deficits in AMPA receptor-mediated excitation of feed-forward inhibition (FFI) in the CTC, on absence seizure generation. AMPA receptors are glutamate activated ion channels and are responsible for most of the fast excitatory synaptic transmission throughout the CNS. In the stargazer mouse model of absence epilepsy, the genetic mutation is in stargazin, a transmembrane AMPA receptor trafficking protein (TARP). This leads to a defect in AMPA receptor insertion into synapses in parvalbumin-containing (PV+) inhibitory interneurons in the somatosensory cortex and thalamus. Mutation in the *Gria4* gene, which encodes for the AMPA receptor subunit GluA4, the predominant AMPA receptor subunit in cortical and thalamic PV + interneurons, also leads to absence seizures. This review explores the impact of glutamatergic synapse dysfunction in the CTC network on absence seizure generation. It also discusses the cellular and molecular mechanisms involved in the pathogenesis of childhood absence epilepsy.

## Introduction

Childhood absence epilepsy (CAE) is classified as a genetic, generalized type of pediatric epilepsy, which is non-convulsive (Scheffer et al., 2017). It occurs in early childhood (peak onset is between 4–10 years) and accounts for approximately 18% of epilepsy in school-aged children. Absence seizures are characterized by sudden, brief impairment of consciousness, accompanied by behavioral arrest. Loss of awareness and unresponsiveness is manifested as vacant episodes (termed absences) during which the child appears to be staring into space. Typical absence seizures are brief lasting 3–20 s but can occur multiple times a day and thus severely impact learning. They appear on electroencephalogram (EEG) as bilaterally synchronous spike and wave discharges (SWDs) at approximately 2.5–4 Hz. Absence seizures, formerly known as “*petit mal*” seizures, were until recently thought to be relatively benign due to their non-convulsive nature and high incidence of remission during childhood and early adulthood (Fisher et al., 2017). However, it is now known that absence seizures in children are also accompanied by comorbid conditions (Tellez-Zenteno et al., 2007). Anxiety and depression are the most frequent comorbidities (Sarkisova et al., 2017). Cognitive, behavioral, and psychiatric comorbidities, including attention deficit hyperactivity disorder, intellectual disability, autism spectrum disorder, depression, unstable mood, and suicidal tendencies are reported in 11–40% of children affected by epilepsy (Austin et al., 2011; Reilly et al., 2014; Terra et al., 2014; Quvile and Wilmshurst, 2016). Furthermore, there is also evidence of morphological changes during development in the cortex of some CAE patients compared to healthy controls, which could affect cognitive abilities (Tosun et al., 2011).

### Seizures and Treatment

Seizures are caused by disruption of the normal excitatory/inhibitory (E/I) balance within brain networks resulting in hyperexcitation. However, the precise cellular and molecular events that transform normal brain circuits into epileptic circuits and the mechanisms that generate seizures in different types of epilepsy are still unclear. Although seizures can be controlled in many patients using anti-epileptic drugs (AEDs), there are often severe side-effects and one-third of patients will continue to have uncontrolled seizures because current AEDs don’t work for them. AEDs even aggravate seizures in some cases (Glauser et al., 2013). As epilepsy is a spectrum disorder, it presents uniquely in each patient, so a “one size fits all” approach to treatment does not work. The variability in response to drug treatment and disease outcome in children with CAE suggests that complex and multifactorial mechanisms may underlie absence seizure generation in patients from different genetic backgrounds. Deciphering the potential mechanisms involved in generation of absence seizures is important for future identification of novel therapeutic targets with higher efficacy for patient-specific treatment.

### The Stargazer Model of Absence Epilepsy

Several rodent models have proved invaluable in studying the cellular and molecular mechanisms underlying absence epilepsy. There appear to be multiple mechanisms through which absence seizures can be generated; with altered glutamatergic excitation implicated in epileptogenesis in many experimental models. Alterations in the expression and function of alpha-amino-3-hydroxy-5-methyl-4-isoxazolepropionic acid (AMPA) receptors, which mediate most of the fast excitatory glutamatergic synaptic transmission in the brain, have been implicated in some models. The stargazer mouse, in particular, has been the focus of studies to understand how a genetic defect resulting in AMPAR deficits at synapses, contributes to the generation of absence seizures. The mutation underlying the epileptic phenotype in stargazers was first identified as a defect in the voltage-dependent calcium channel (VDCC) γ2 subunit gene, *Cacng2* (Noebels et al., 1990; Letts et al., 1998). This severely reduces the normal expression of the γ2 subunit protein called stargazin. Stargazin is involved in trafficking, synaptic targeting and modulation of AMPA receptors at excitatory synapses (Hashimoto et al., 1999; Chen et al., 2000; Tomita et al., 2003, 2005; Nicoll et al., 2006). It belongs to a family of transmembrane AMPA receptor regulatory proteins (TARPs) which are differentially expressed in different brain regions and neurons (Tomita et al., 2003). It was the first TARP to be identified; and was named TARP-γ2, due to its homology to the γ1 subunit of skeletal muscle VDCCs. Absence of stargazin (TARP-γ2) in the stargazer mutant mouse leads to absence epilepsy and cerebellar ataxia (Noebels et al., 1990). Absence seizures are known to originate from disturbance within the corticothalamocortical (CTC) network. Stargazin expression in the cortex and thalamus is limited to inhibitory gamma-aminobutyric acid (GABA) interneurons (Tao et al., 2013), and specifically to parvalbumin containing (PV +) GABAergic interneurons (Maheshwari et al., 2013).

### The Corticothalamocortical Network

**FIGURE 1:**
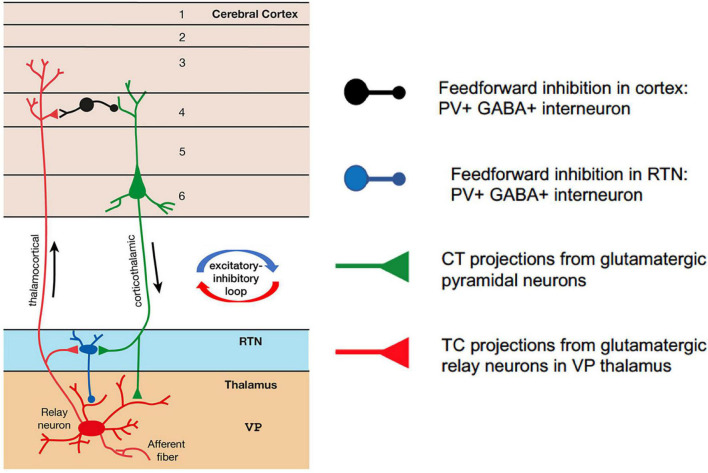
Simplified schematic of the corticothalamocortical (CTC) network in the rodent brain. Relay neurons in the ventral posterior (VP) thalamus are reciprocally connected with the pyramidal neurons in the cortex. Feedforward PV + inhibitory interneurons in the reticular thalamic nucleus (RTN) project onto the relay neurons and are excited at AMPA synapses by corticothalamic projections from pyramidal cells. Feedforward PV + inhibitory interneurons in the cortex are excited at AMPA synapses by thalamocortical projections from relay neurons in VP thalamus.

The CTC network comprises reciprocal connections between the thalamus and the cortex (Figure 1). In normal functioning of the CTC network, the thalamus receives sensory information from the periphery. Glutamatergic relay neurons within the ventral posterior (VP) thalamic nucleus send excitatory thalamocortical (TC) projections to glutamatergic pyramidal cells in layer IV of the cortex. The pyramidal cells in turn send corticothalamic (CT) projections back to the relay neurons in the thalamus from cortical layers V/VI, which are the output layers of the cortex (Jones, 1998; Constantinople and Bruno, 2013). Both the TC and CT projections also send collateral branches into the reticular thalamic nucleus (RTN), which forms a thin sheath of inhibitory GABAergic PV + interneurons that surrounds the thalamic relay nuclei. These CT and TC projections to the RTN are reciprocally connected, allowing the RTN to evaluate the sensory information transmitted to-and-from the cortex. The RTN inhibitory interneurons do not project out of the thalamus; they send feed-forward inhibition (FFI) to the thalamic relay neurons. In this way, the RTN plays an important role in regulating the excitability of thalamic relay cells (Bal et al., 1995; Sohal and Huguenard, 1998). CT collateral activation of RTN feed-forward inhibitory neurons is much stronger than direct CT activation of relay neurons in the VP, therefore cortical activation is mainly inhibitory *via* the collateral RTN pathway (Destexhe et al., 1998; Golshani et al., 2001; Beenhakker and Huguenard, 2009).

The SWDs, which are the hallmark of absence seizures on EEG, arise from aberrant hypersynchronous activity within this network (Snead, 1995; McCormick and Contreras, 2001). However, the precise cellular and molecular mechanisms underlying the genesis of absence seizures are still largely unknown and appear multifactorial. There are potentially different microcircuits within the CTC network that could be dysfunctional in different patients, hence accounting for the variability in response to drug treatment. It is critical to understand the microcircuit and neuron-specific mechanisms that underlie generation of absence seizures, which arise from different genetic backgrounds, in order to identify novel therapeutic targets for treatment of this type of seizure.

### Feed-Forward Inhibition in the Corticothalamocortical Network

Research using the stargazer mouse model of absence epilepsy (Barad et al., 2012, 2017; Seo and Leitch, 2014, 2015, 2017; Adotevi and Leitch, 2016, 2017, 2019) has demonstrated that region-specific alterations in AMPA receptor expression in inhibitory microcircuits within the CTC network may be a key factor contributing to pathological hypersynchronous oscillatory activity in some forms of absence epilepsy. Specifically, a selective decrease in AMPA receptor expression at excitatory input synapses on RTN inhibitory interneurons from CT afferent projections (CT-RTN) but not at excitatory input synapses onto VP relay neurons (CT-VP) has been identified by Barad et al. (2012). As the inhibitory interneurons in RTN provide FFI to relay neurons in the VP, loss of AMPA receptors at CT-RTN synapses could lead to a loss of FFI within this microcircuit. A similar loss of AMPARs within inhibitory feed-forward microcircuits in the somatosensory cortex has also been reported (Adotevi and Leitch, 2016, 2017, 2019). FFI is essential to prevent runaway excitation within the CTC network. Epilepsy is caused by disruption of the normal excitatory-inhibitory (E/I) balance within brain networks, resulting in hyperexcitation and seizures. GABAergic feed-forward interneurons play a crucial role in the prevention of seizures by regulating this delicate E/I balance. A specific loss in CT-RTN excitation, leading to impaired FFI of thalamic relay nuclei, has also been demonstrated in the absence epileptic Gria4 knockout mouse, which lacks the AMPAR GluA4 subunit (Paz et al., 2011). GluA4-AMPARs are more abundant in RTN neurons than on TC relay cells (Golshani et al., 2001). Loss of GluA4 expression in the Gria4^–/–^ mouse (Beyer et al., 2008) resulted in a selective impairment in CT-RTN firing, but not in CT-relay neuron or feedback relay neuron-RTN synaptic function (Paz et al., 2011). Collectively, these findings suggest that impairment of thalamic FFI due to weakened AMPAR-mediated excitatory input to inhibitory RTN neurons may contribute to seizures in these two absence epilepsy mouse models.

### Impact of Silencing Feed-Forward Inhibition Using Designer Receptors Exclusively Activated by Designer Drug Technology

To test whether loss of FFI is directly related to the generation and maintenance of absence seizures, Panthi and Leitch (2019) used Designer Receptors Exclusively Activated by Designer Drug (DREADD) technology to selectively silence PV + interneurons in the CTC network. DREADD technology involves insertion of engineered receptors (designer receptors) that are only activated by synthetic ligands (designer drugs) into specific neurons (Roth, 2016). DREADD receptors are mutated muscarinic G-protein receptors, which are either excitatory (hM3Dq) or inhibitory (hM4Di). They can be inserted into transgenic mice *in vivo* using viral vector methods (Zhu et al., 2014; Urban and Roth, 2015). Alternatively, strains of DREADD mice can be used that express either the activating Gq-DREADD or inhibiting Gi-DREADD, under the control of a strong ubiquitous promoter, which is separated from the DREADD by a loxP site-flanked Stop signal. Mating of these strains with any Cre-driver mouse line removes the Stop signal only in the cell type specified by the Cre-driver used. This cell-specific DREADD can then be activated by injection or oral application of the designer drug i.e., clozapine-N-oxide (CNO) (Zhu et al., 2016). This later method was used by Panthi and Leitch (2019) to express inhibiting Gi-DREADDs in PV + interneurons. They found that inactivating FFI (either in the somatosensory cortex or the RTN thalamus by focal injections of CNO) generates absence-like SWDs in normal non-epileptic mice. Furthermore, selectively activating PV + inhibitory interneurons within the CTC network during chemically-induced absence seizures, was sufficient to prevent or reduce seizure activity (Panthi and Leitch, 2021). In contrast, focal injection of CNO into either the somatosensory cortex or RTN thalamus of non-DREADD wildtype control animals, had no effect on chemical-induced absence seizures. These data demonstrate a potential for targeting FFI interneurons within the CTC network in future therapeutic strategies to control seizures in some cases of human absence epilepsy.

## Conclusion

This review highlights the impact of glutamatergic synapse dysfunction in the CTC network on absence seizure generation. Loss of FFI in CTC microcircuits, as a result of a mutation in the AMPA receptor trafficking protein stargazin, results in SWDs. Hence intervention strategies to regulate the activation of specific inhibitory interneurons within this network could be a potential seizure suppressing mechanism in some absence epilepsy patients. Suppression of epileptiform activity by modifying the synaptic output from specific inhibitory interneurons through the use of DREADD and optogenetic technologies is now possible (Krook-Magnuson and Soltesz, 2015). Furthermore, these approaches can be used to investigate any comorbidity between seizures and behavioral changes related to neurological and neuropsychiatric disorders.

## Author Contributions

The author confirms being the sole contributor of this work and has approved it for publication.

## Conflict of Interest

The author declares that the research was conducted in the absence of any commercial or financial relationships that could be construed as a potential conflict of interest.

## Publisher’s Note

All claims expressed in this article are solely those of the authors and do not necessarily represent those of their affiliated organizations, or those of the publisher, the editors and the reviewers. Any product that may be evaluated in this article, or claim that may be made by its manufacturer, is not guaranteed or endorsed by the publisher.
